# Genomic Evolution of Two *Acinetobacter baumannii* Clinical Strains from ST-2 Clones Isolated in 2000 and 2010 (ST-2_clon_2000 and ST-2_clon_2010)

**DOI:** 10.1128/genomeA.01182-16

**Published:** 2016-10-20

**Authors:** M. López, A. Rueda, J. P. Florido, L. Blasco, E. Gato, L. Fernández-García, L. Martínez-Martínez, F. Fernández-Cuenca, J. Pachón, J. M. Cisneros, J. Garnacho-Montero, J. Vila, J. Rodríguez-Baño, A. Pascual, G. Bou, M. Tomás

**Affiliations:** a Department of Microbiology, Complejo Hospitalario Universitario, A Coruña, INIBIC, La Coruña, Spain; b Bioinformatics, GBPA, Seville, Spain; c Department of Clinical Microbiology, Hospital Universitario Marqués de Valdecilla-IFIMAV, Santander, Spain; d Departament of Molecular Biology, University of Cantabria, Santander, Spain; e Unidad Intercentros de Enfermedades Infecciosas, Microbiología y Medicina Preventiva, Hospital Universitario Virgen Macarena, Seville, Spain; f Institute of Biomedicine of Seville (IBiS), University Hospital Virgen del Rocío/CSIC/University of Seville, Seville, Spain; g Department of Clinical Microbiology, Hospital Clínic, School of Medicine, University of Barcelona, Barcelona and ISGlobal, Barcelona Centre for International Health Research (CRESIB), Hospital Clínic, Barcelona, Spain; h Spanish Network for the Research in Infectious Diseases (REIPI RD12/0015), Seville, Spain

## Abstract

*Acinetobacter baumannii* is a successful nosocomial pathogen due to its ability to persist in hospital environments by acquiring mobile elements such as transposons, plasmids, and phages. In this study, we compared two genomes of *A. baumannii* clinical strains isolated in 2000 (ST-2_clon_2000) and 2010 (ST-2_clon_2010) from GenBank project PRJNA308422.

## GENOME ANNOUNCEMENT

*Acinetobacter baumannii* is a successful nosocomial pathogen, especially in intensive care units (ICUs) (1, 2). This is due to the pathogen’s ability to persist in the hospital environment for long periods of time by acquiring mobile genomic elements (transposons, plasmids, and phages) that are the main driving forces for the genome (3).

In this study, we sequenced two *A. baumannii* clinical strains (ST-2_clon_2000 and ST-2_clon_2010) from the GEIH-REIPI Spanish Multicenter *Acinetobacter baumannii* Study II (2000–2010) (GenBank accession no. PRJNA308422). Strain ST-2_clon_2000 (susceptible to carbapenems) compared with ST-2_clon_2010 (resistant to carbapenems) showed a clonal relation of 90% by pulsed-field gel electrophoresis, after a decade in the hospital environment (in the same ICU).

Next-generation sequencing of both strains was performed with a Roche 454 GS FLX+ sequencer according to the manufacturer’s instructions (Roche 454 Life Sciences, Branford, CT, USA). Reads were assembled with Newbler. Putative open reading frames were predicted from assembled contigs with GeneMarKS (4), previously trained with the *A. baumannii* genome (GI:83207914). Functional annotation of each predicted protein was carried out with Blast2Go (5) and RAST (6). The rRNA and tRNA were identified using RNAmmer (7) and tRNAscan-SE version 1.21 (8). The genomes of ST-2_clon_2000 and ST-2_clon_2010 were compared using Mauve (9).

ST-2_clon_2000 read assemblies generated 64 contigs, accounting for a size of 3,991,758 bp (mean coverage of 36.49-fold). BLAST analysis showed that 62 contigs were of chromosomal origin (3,922,379 bp) and that two contigs were plasmidic sequences with high similarity to *A. baumannii* TCDC-AB0715 plasmid p2ABTCDC0715 (6,650 bp). Assembly of the ST-2_clon_2010 reads produced 77 contigs representing 4,092,613 bp (mean coverage of 40.38-fold). BLAST similarity searches revealed that 74 contigs were part of the genomic chromosome (4,013,020 bp), two contigs were plasmidic with high similarity to *A. baumannii* TCDC-AB0715 plasmid p2ABTCDC0715 (70,004 bp), and one contig was a whole plasmid with very high similarity to *A. baumannii* pMMCU3 plasmid with the *bla*_OXA-24_ gene and AbkA/AbkB toxin/antitoxin system) (10, 11).

Annotation of strain ST-2_clon_2000 predicted 3,759 protein-coding sequences, 5S, 16S, and 23S rRNA genes, and 63 tRNAs, whereas annotation of ST-2_clon_2010 predicted 3,923 protein-coding sequences, 5S, 16S, and 23S rRNA genes, and 63 tRNAs.

Comparison of chromosomal sequences of strains ST-2_clon_2000 and ST-2_clon_2010 revealed the following: (i) 3,627 proteins were identical in both strains; (ii) 88 proteins were very similar; (iii) five proteins shared a similarity of less than 60%; (iv) 20 proteins were unique to ST-2_clon_2000; (v) 212 proteins were only present in ST-2_clon_2010, most of which were similar to phage proteins; and (vi) the chromosome of strain ST-2_clon_2010 contains several bacteriophage sequences.

Finally, comparison of plasmidic sequences indicated the following: (i) 90 proteins were identical in both strains; (ii) three proteins showed a high degree of similarity (>60% similarity); (iii) 17 plasmidic proteins were found only in ST-2_clon_2010 (pMMCU3 harboring the *bla*_OXA-24_ gene and AbkA/AbkB toxin/antitoxin system) (10, 11); and (iv) ST-2_clon_2000 did not share any plasmidic proteins with ST-2_clon_2010.

### Accession number(s).

The ST-2_clon_2000 whole-genome shotgun (WGS) project has been deposited in DDBJ/ENA/GenBank under the accession number LJHA00000000. The version described in this paper is version LJHA01000000 and consists of sequences LJHA01000001 to LJHA01000064. The ST-2_clon_2010 WGS project has been deposited in DDBJ/ENA/GenBank under the accession number LJHB00000000. The version described in this paper is version LJHB01000000 and consists of sequences LJHB01000001 to LJHB01000077. Both WGS studies belong to the GEIH-REIPI Spanish Multicenter *Acinetobacter baumannii* Study II 2000–2010 project PRJNA308422.
